# Prognostic Value of Hematologic Indices and Composite Models in Anal Squamous Cell Carcinoma Treated with Image-Guided Chemoradiotherapy

**DOI:** 10.3390/cancers17233838

**Published:** 2025-11-29

**Authors:** Soňa Argalácsová, Petr Dytrych, Monika Wágnerová, Vladimír Černý, Jan Špaček, Stanislav Hloušek, Pavel Koželský, Jakub Tesař, David Hoskovec, Michal Vočka

**Affiliations:** 1 Department of Oncology, General University Hospital in Prague and First Faculty of Medicine, Charles University, 128 08 Prague, Czech Republic; 2 1st Department of Surgery, General University Hospital in Prague and First Faculty of Medicine, Charles University, 128 08 Prague, Czech Republic; petr.dytrych@vfn.cz (P.D.); david.hoskovec@vfn.cz (D.H.); 3 Department of Radiology, General University Hospital in Prague and First Faculty of Medicine, Charles University, 128 08 Prague, Czech Republic

**Keywords:** anal canal neoplasms, carcinoma, squamous cell, chemoradiotherapy, risk stratification, image-guided radiotherapy, prognosis, neutrophil-to-lymphocyte ratio, platelet-to-lymphocyte ratio

## Abstract

Background: Anal squamous cell carcinoma (ASCC) is a rare malignancy primarily treated with chemoradiotherapy (CRT). This study evaluated outcomes and the prognostic value of simple hematologic indices in patients receiving modern image-guided CRT. Methods: Fifty-five patients with non-metastatic ASCC treated between 2017 and 2025 were retrospectively analyzed. Survival was estimated by Kaplan–Meier methods, and prognostic factors were assessed by log-rank testing and Cox regression. Baseline neutrophil-to-lymphocyte (NLR) and platelet-to-lymphocyte (PLR) ratios were analyzed individually and in combination with nodal status. Results: At a median follow-up of 53.1 months, overall survival reached 90% at 5 years, whereas disease-free survival declined to 51%. Nodal positivity showed a non-significant trend toward poorer DFS. Baseline PLR ≥ 150 was significantly associated with inferior DFS in univariable analysis (HR 5.28, 95% CI 1.12–24.97, *p* = 0.036), while NLR ≥ 3 showed a borderline effect (*p* = 0.108). In multivariable models, PLR retained borderline prognostic relevance (*p* = 0.083), whereas Kaplan–Meier curves indicated non-significant trends (*p* = 0.129 and 0.055). Integrated models combining nodal status with PLR ± NLR improved risk discrimination: Model A (N + PLR ≥ 150 ± NLR ≥ 3) showed a trend (*p* = 0.059), and Model B (N + PLR ≥ 150) reached significance (*p* = 0.021; C-index ≈ 0.68–0.69). Conclusions: Modern CRT achieved excellent OS with acceptable toxicity, though early recurrences limited DFS. Integrating hematologic indices with nodal status provides a pragmatic, cost-effective approach for individualized risk assessment and follow-up in ASCC.

## Simple Summary

Definitive chemoradiotherapy remains the cornerstone of treatment for anal squamous cell carcinoma (ASCC), achieving excellent overall survival in most patients. However, disease-free survival is often limited by early recurrences within the first three years after treatment. In this single-center cohort, nodal positivity showed a consistent trend toward poorer outcomes, while baseline hematologic indices—particularly a platelet-to-lymphocyte ratio (PLR ≥ 150)—provided additional prognostic information. When combined with nodal status, these simple laboratory markers enabled effective stratification of disease-free survival into low-, intermediate-, and high-risk groups. The proposed composite models are inexpensive, reproducible, and based on routinely available data, allowing straightforward implementation in everyday oncology practice. Integrating such models into follow-up algorithms could help personalize surveillance intensity, identify patients requiring closer monitoring, and support stratification in future clinical trials.

## 1. Introduction

Anal squamous cell carcinoma (ASCC) is an uncommon malignancy, accounting for approximately 1–2% of all gastrointestinal cancers. According to the GLOBOCAN 2022 database of the International Agency for Research on Cancer (IARC), an estimated 54,306 new cases and 22,035 deaths worldwide, corresponding to an age-standardized incidence rate of 0.54 per 100,000 population [1]. Incidence has been rising in many high-income countries, particularly among women and older adults [2]. In the Czech Republic, ASCC remains rare, with an incidence of 1.5–2.0 per 100,000 and mortality of 0.7–0.8 per 100,000; trends over recent years have been stable to slightly increasing [3,4].

Definitive concurrent chemoradiotherapy (CRT) combining 5-fluorouracil (5-FU) or oral capecitabine with mitomycin C (MMC) has been the standard of care since the late 1980s. Early randomized trials, including UKCCCR ACT I and EORTC 22861, demonstrated that CRT significantly improved locoregional control and colostomy-free survival compared with radiotherapy (RT) alone [5,6]. Subsequent phase III studies confirmed the superiority of MMC-based CRT: RTOG 98-11 showed inferior outcomes with cisplatin-based regimens [7], while ACT II demonstrated no added benefit of cisplatin or maintenance chemotherapy [8]. The introduction of modern intensity-modulated and image-guided radiotherapy (IMRT/IGRT) has further improved dose conformity and toxicity profiles, although local recurrence remains the predominant pattern of failure [9,10].

Despite decades of refinement, several questions remain unresolved. These include the optimal radiosensitizing regimen (MMC vs. cisplatin), whether capecitabine can fully replace 5-FU, and the appropriate extent of elective inguinal irradiation [11]. Moreover, treatment de-escalation in early-stage anal squamous cell carcinoma has gained interest but remains investigational [12].Prognosis in ASCC is primarily determined by tumor stage, nodal involvement, and treatment-related factors [13]. Viral and molecular markers, particularly HPV and p16 positivity, are associated with improved outcomes [14]. However, growing evidence indicates that host-related factors—especially systemic inflammation—also influence tumor progression and treatment response. Simple blood-derived markers such as the neutrophil-to-lymphocyte (NLR) and platelet-to-lymphocyte (PLR) ratios reflect the balance between protumor inflammatory drive and adaptive immune control. These markers have shown prognostic value in ASCC and other malignancies [15]. Beyond single inflammatory markers, several composite indices have recently been proposed. Casadei-Gardini et al. introduced the Anal Cancer Response Classifier (ARC), which integrates inflammatory and clinical variables to predict outcomes after CRT [16]. Similarly, Rimini et al. introduced the Hemo-Eosinophil Inflammation (HEI) index, later externally validated by Franco et al., demonstrating significant prognostic discrimination for disease-free and overall survival [17,18]. In parallel, the systemic immune-inflammation index (SII)—calculated from neutrophil, platelet, and lymphocyte counts—has emerged as a pragmatic indicator of systemic inflammatory status, with elevated values associated with inferior outcomes across multiple solid tumors and in early ASCC cohorts [16,17,18].

Given the increasing use of image-guided radiotherapy and growing interest in systemic inflammatory markers, contemporary real-world data are needed to clarify their prognostic value in ASCC. This study therefore aimed to evaluate survival, toxicity, and recurrence patterns in patients treated with modern IMRT/IGRT and to assess the prognostic utility of baseline hematologic indices, alone and in combination with nodal status, in predicting disease-free survival.

## 2. Materials and Methods

### 2.1. Study Design and Patients

We conducted a retrospective, single-center cohort study including consecutive patients with histologically confirmed anal squamous cell carcinoma (ASCC) who received definitive chemoradiotherapy (CRT) at the General University Hospital in Prague between January 2017 and May 2025.

Eligible patients had non-metastatic disease (stage I–III according to the 8th edition of the American Joint Committee on Cancer [AJCC] staging system), received curative-intent external-beam radiotherapy with concurrent chemotherapy, and had available baseline clinical and laboratory data.

Patients with distant metastases, prior pelvic radiotherapy, or incomplete treatment were excluded. Follow-up data were collected until 15 September 2025. Demographic, clinical, treatment, laboratory, and follow-up information were obtained from electronic medical records and the institutional cancer registry.

### 2.2. Treatment Protocols

External-beam radiotherapy (RT) was delivered using image-guided intensity-modulated radiotherapy (IG/IMRT) with a helical tomotherapy system. Daily image guidance with megavoltage computed tomography (MVCT) was performed before each fraction, allowing online setup correction within a tolerance of ±3–5 mm.

Target delineation and dose constraints followed the international consensus guidelines of the European Society for Radiotherapy and Oncology—Advisory Committee on Radiation Oncology Practice (ESTRO ACROP) and the Radiation Therapy Oncology Group (RTOG). Elective pelvic and inguinal nodal regions were included according to institutional policy.

The prescribed dose was typically 50.4–54 Gy in 28–30 fractions to elective volumes and 59.4–60 Gy in 30–33 fractions to gross tumor volumes. Concurrent chemotherapy consisted of mitomycin C (MMC; 10 mg/m^2^ intravenous bolus, days 1 and 29) combined with either infusional 5-fluorouracil (5-FU; 1000 mg/m^2^/day, continuous infusion on days 1–4 and 29–32) or, in patients treated after 2020, oral capecitabine (825 mg/m^2^ twice daily on radiotherapy days). Dose modifications were permitted in cases of clinically significant toxicity.

### 2.3. Definitions

The co-primary outcomes were overall survival (OS) and disease-free survival (DFS).

OS was defined as the time from the completion of radiotherapy (RT) to death from any cause.

DFS was defined as the time from the completion of RT to the first documented recurrence (local, regional, or distant) or death from any cause, whichever occurred first.

Patients without an event were censored at the date of last follow-up (data cutoff: 15 September 2025). Patterns of failure were categorized as local (anorectal), regional (pelvic or inguinal lymph nodes), or distant metastases.

Secondary outcomes included acute and late toxicities, graded according to the Common Terminology Criteria for Adverse Events (CTCAE), version 5.0. Acute toxicity was defined as occurring within ≤90 days from the start of RT, and late toxicity as occurring >90 days from RT completion.

Exploratory analyses evaluated the prognostic significance of baseline hematologic indices—specifically the neutrophil-to-lymphocyte ratio (NLR) and platelet-to-lymphocyte ratio (PLR). These were analyzed both as predefined dichotomies (NLR ≥ 3, PLR ≥ 150) and as log-transformed, standardized continuous variables (per 1-SD increase).

Composite clinical–hematologic models integrating nodal status (N0 vs. N+), T stage, and hematologic indices were additionally tested.

Finally, the performance of these models was compared to contextualize their prognostic value with published inflammation-based classifiers, namely the Hemo-Eosinophil Inflammation (HEI) index and an approximation of the Anal Cancer Response Classifier (ARC). At baseline, no patient had a documented acute infection, active hematologic disorder, or ongoing systemic corticosteroid treatment, minimizing potential confounding effects on inflammatory markers.

### 2.4. Follow-Up

Patients were scheduled for follow-up visits every 3–4 months during the first 3 years and every 6–12 months thereafter. Survival status was confirmed through review of medical records and national registries.

### 2.5. Risk Stratification Models and Comparative Analysis

Based on clinical relevance and the results of univariable and multivariable analyses, we defined two simple three-tier risk models integrating nodal status with baseline inflammatory indices (literature-based cut-offs: PLR ≥ 150; NLR ≥ 3). Model A combined nodal status (N0 vs. N+) with both platelet-to-lymphocyte ratio (PLR) and neutrophil-to-lymphocyte ratio (NLR). One point was assigned for each unfavorable feature (N+, PLR ≥ 150, NLR ≥ 3); the total score (0–3) was grouped as low = 0, intermediate = 1, and high = 2.

Model B combined nodal status with PLR only, assigning one point for N+ and one for PLR ≥ 150; the sum was grouped as low = 0, intermediate = 1, and high = 2.

Disease-free survival (DFS) across risk groups was analyzed using Kaplan–Meier estimates with global log-rank testing. Model discrimination and overall fit were quantified by Harrell’s concordance index (C-index) and the Akaike Information Criterion (AIC), respectively.

To benchmark the proposed models, their prognostic performance was compared with published inflammation-based classifiers, namely the Hemo-Eosinophil Inflammation (HEI) index—defined as the sum (range 0–3) of hemoglobin < 12 g/dL, systemic immune-inflammation index (SII) > 560, and eosinophil count ≥ 100/µL, categorized as low (0–1) or high (2–3)—as well as an approximation of the Anal Cancer Response Classifier (ARC), reconstructed from its original components (SII, nodal status, and hemoglobin level).

### 2.6. Statistical Analysis

Baseline and treatment characteristics were summarized descriptively. Overall survival (OS) and disease-free survival (DFS) were estimated using the Kaplan–Meier method, and 1-, 3-, and 5-year survival rates were reported with corresponding 95% confidence intervals (CI). Median follow-up was calculated using the reverse Kaplan–Meier method. Prognostic factors were assessed using univariable and multivariable Cox proportional-hazards models; the proportional-hazards assumption was checked by Schoenfeld residuals.

Two pre-specified three-tier risk models integrating nodal status with baseline inflammatory indices were evaluated using literature-based cut-offs (PLR ≥ 150; NLR ≥ 3). Model A combined nodal status (N0 vs. N+) with both platelet-to-lymphocyte ratio (PLR) and neutrophil-to-lymphocyte ratio (NLR): one point was assigned for each unfavorable feature (N+, PLR ≥ 150, NLR ≥ 3); the total score (0–3) was grouped as low = 0, intermediate = 1, high = 2. Model B combined nodal status with PLR only: one point for N+ and one for PLR ≥ 150; the sum (0–2) was grouped as low = 0, intermediate = 1, high = 2. For each model, DFS separation across groups was evaluated with Kaplan–Meier curves and global log-rank tests. For DFS analyses, any recorded disease recurrence or progression (dataset codes 1–5) was treated as an event (DFS = 1), whereas code 0 indicated censoring. Discrimination and overall fit were quantified by Harrell’s concordance index (C-index) and the Akaike Information Criterion (AIC), respectively; where indicated, C-index uncertainty was summarized by bootstrap resampling (B = 1000).

Continuous inflammatory markers (e.g., NLR, PLR) were analyzed both as predefined dichotomies (NLR ≥ 3, PLR ≥ 150) and as log-transformed standardized variables (per 1-SD increase) in the Cox models.

All statistical analyses were performed in R (version 4.5.1; R Foundation for Statistical Computing, Vienna, Austria) using the survival, survminer, rms, timeROC, and pec packages within RStudio version 2025.01.0 (Posit). The random seed was set to 12345. Two-sided *p*-values < 0.05 were considered statistically significant.

### 2.7. Ethical Considerations

The study was conducted in accordance with the principles of the Declaration of Helsinki. Ethical approval for the analysis and publication of anonymized patient data was obtained from the Ethics Committee of the General University Hospital in Prague (No. 146/25 S-IV, 20 October 2025). Owing to the retrospective nature of the study, the requirement for individual informed consent for this analysis was waived in accordance with national regulations. All patients had previously provided written informed consent for chemoradiotherapy, and all treatments were delivered in compliance with current national and international standards of care.

## 3. Results

### 3.1. Patient and Treatment Characteristics

A total of 55 patients with histologically confirmed anal squamous cell carcinoma (ASCC) treated between January 2017 and May 2025 were included in the analysis. The median age at diagnosis was 63 years (range, 37–78 years), and 78% of the patients were female. Most presented with stage II–III disease, with T2–T3 tumors predominating. Nodal involvement was observed in more than two-thirds of the cohort.

All patients received definitive chemoradiotherapy with 5-fluorouracil and mitomycin C. Radiotherapy was delivered using image-guided helical tomotherapy, according to one of three institutional planning protocols: simultaneous integrated boost (SIB, 44%), RTOG-based (35%), or sequential boost (22%). The median overall treatment time (OTT) was 42 days (range, 35–74 days).

An overview of baseline demographic, clinical, and treatment characteristics is summarized in Table 1.

### 3.2. Survival Analysis

At a reverse Kaplan–Meier median post-radiotherapy follow-up of 53.1 months (95% CI 34.7–58.0), six deaths and 19 disease-free survival (DFS) events were observed among 55 patients. Of the six deaths, four were disease-related, while two resulted from non-disease-related causes (one cardiac failure shortly after therapy and one progression of metachronous malignancy).

**Figure 1:**
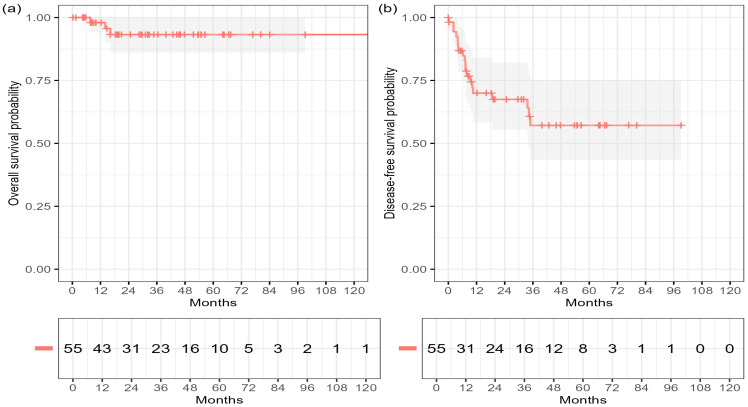
Overall and disease-free survival after definitive chemoradiotherapy for anal squamous cell carcinoma (ASCC). Kaplan–Meier curves show (**a**) overall survival (OS) and (**b**) disease-free survival (DFS). Shaded areas indicate 95% confidence intervals; numbers at risk are shown below each plot.

The estimated overall survival (OS) rates were 96.2% at 12 months (95% CI 86.3–99.0) and 90.0% at 36 and 60 months (95% CI 76.8–95.9). Median OS was not reached. The corresponding DFS rates were 68.0% at 12 months (95% CI 53.1–79.1), 55.6% at 36 months (95% CI 39.0–69.4), and 51.0% at 60 months (95% CI 35.8–66.1), with a median DFS of 84.0 months (Figure 1, Table 2).

During follow-up, 19 patients (34.5%) experienced disease recurrence after chemoradiotherapy. Local recurrence at the primary site was the most frequent pattern (9 patients, 47%), followed by pulmonary (32%), hepatic (16%), and other distant sites (26%). Combined local and distant failures occurred in five patients (26%). The median time to recurrence was 7.3 months (IQR 4.0–10.0; range 2.3–35 months) (Table 3).

Due to the limited number of OS events, inferential modeling for OS was considered exploratory. Prognostic modeling for DFS is detailed in Section 3.4.

### 3.3. Toxicity

Acute and late toxicities were assessed according to the Common Terminology Criteria for Adverse Events (CTCAE), version 5.0. Systematic documentation of late non-hematologic toxicities was less consistent in older records of this retrospective cohort; therefore, these findings should be interpreted with caution.

Acute toxicities were common, predominantly involving skin reactions (96%, grade 3 in 36%) and hematologic events (87%, grade 3–4 in 47%). No severe (grade ≥ 3) gastrointestinal or genitourinary toxicity was observed.

Late adverse events were mostly grade 1–2, with only a few grade ≥ 3 occurrences (one gastrointestinal, one genitourinary, and three hematologic). Two patients were non-evaluable for late toxicity due to early non-disease-related deaths shortly after radiotherapy. Over the past two years, enhanced prophylactic skin care has contributed to later onset, shorter duration, and improved recovery of acute dermatologic reactions.

These findings are broadly consistent with contemporary IMRT/IGRT series, which have reported lower rates of severe non-hematologic toxicity compared with historical 3D-CRT, while grade 3–4 hematologic events remain relatively frequent during concurrent chemoradiotherapy [19,20,21,22,23,24].

A detailed distribution of acute and late toxicities is summarized in Table 4.

### 3.4. Prognostic Factors

Univariable Cox regression for DFS identified nodal positivity (N+) as a factor showing a trend toward an increased risk of recurrence (HR 2.79, 95% CI 0.81–9.66; *p* = 0.105), whereas T stage, HPV status, tumor grade, and age were not significantly associated with outcome.

In the multivariable model including T and N stage, nodal status again showed a non-significant trend toward worse DFS (HR 2.74, 95% CI 0.74–10.10; *p* = 0.130), whereas T stage was not significant (HR 1.05, 95% CI 0.40–2.74; *p* = 0.925). These results are summarized in Table 5.

**Figure 2:**
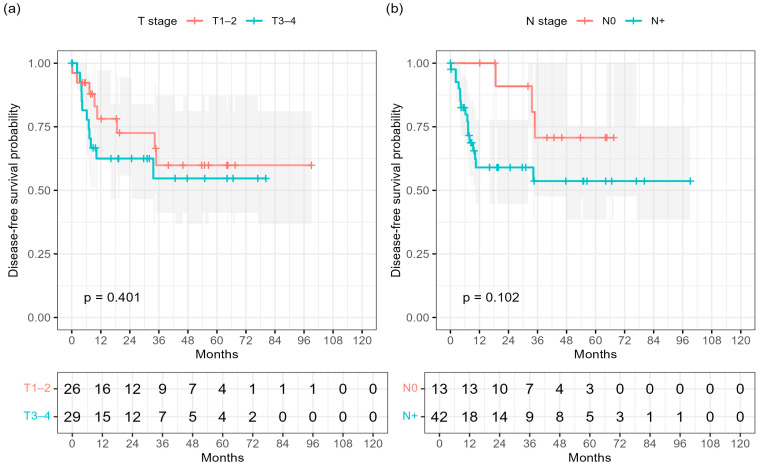
Disease-free survival (DFS) according to baseline subgroups: (**a**) T stage (T1–2 vs. T3–4) and (**b**) nodal status (N0 vs. N^+^). Log-rank *p*-values are shown.

Consistently, Kaplan–Meier analysis demonstrated inferior DFS in node-positive patients compared with those with node-negative (N0) disease (log-rank *p* = 0.102; Figure 2b). No significant difference was observed for T-stage (log-rank *p* = 0.401; Figure 2a).

Due to the very low number of disease-related deaths (n = 4), Cox regression analyses for OS were not feasible and are therefore not presented in detail. Overall survival, defined as all-cause mortality (six deaths in total), is interpreted descriptively only (see Section 3.2). In this cohort, conventional T and N stages showed only minimal separation of overall survival (Figures S1 and S2; log-rank *p* = 0.484 and *p* = 0.275, respectively), which likely reflects the small number of OS events. In contrast, inflammation-based indices demonstrated substantially stronger discriminatory performance for disease-free survival, where event numbers were sufficient to reveal prognostic signal. 

### 3.5. Subgroup Analyses

Kaplan–Meier subgroup analyses were performed to explore potential differences in DFS according to baseline clinical factors.

Patients with T3–4 tumors showed numerically lower DFS compared with those with T1–2 disease; however, this difference did not reach statistical significance (3-year DFS 54.7% vs. 59.9%; log-rank *p* = 0.401; Figure 2a).

Similarly, nodal involvement was associated with a trend toward inferior DFS compared with node-negative disease (3-year DFS 53.6% vs. 70.7%; *p* = 0.102; Figure 2b).

These results corroborate the trend observed in the multivariable analysis, where nodal status remained the key driver of recurrence risk.

DFS outcomes were comparable across age groups (3-year DFS 58.2% for ≤70 years vs. 54.3% for >70 years; *p* = 0.89) and did not differ by overall treatment time (OTT; 3-year DFS 56.1% for ≤49 days vs. 68.8% for >49 days; *p* = 0.84). Likewise, HPV status showed no significant association with DFS (3-year DFS 59.1% for HPV-positive vs. 52.3% for HPV-negative or unknown; *p* = 0.71).

Subgroup analyses for overall survival (OS) according to T and N stage also did not demonstrate statistically significant differences (log-rank *p* = 0.484 and *p* = 0.275, respectively; Figures S1 and S2), consistent with the limited number of death events.

A detailed summary of subgroup DFS analyses is provided in Table 6. None of the evaluated clinical variables reached statistical significance.

### 3.6. Hematological Indices

Baseline hematologic indices were analyzed as potential prognostic markers for disease-free survival (DFS).

**Figure 3:**
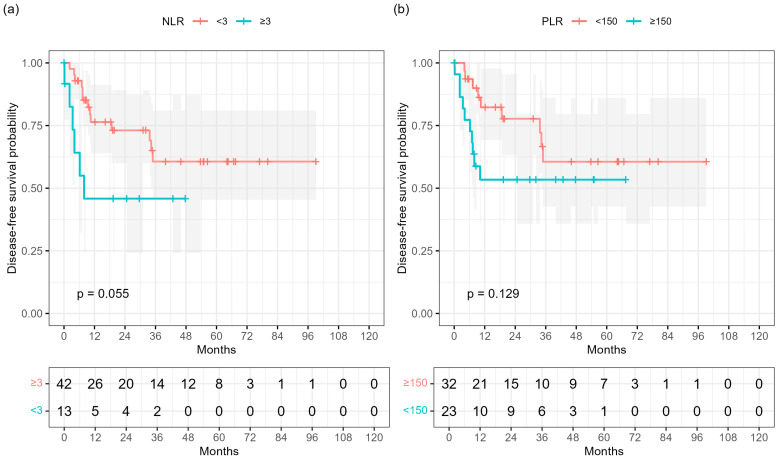
Disease-free survival (DFS) according to baseline hematologic indices. Kaplan–Meier curves show (**a**) neutrophil-to-lymphocyte ratio (NLR) dichotomized at 3 (*p* = 0.055) and (**b**) platelet-to-lymphocyte ratio (PLR) dichotomized at 150 (*p* = 0.129). Shaded areas represent 95% confidence intervals; numbers at risk are shown below each plot.

In Kaplan–Meier analyses, a higher neutrophil-to-lymphocyte ratio (NLR ≥ 3) showed a borderline association with shorter DFS (log-rank *p* = 0.055; Figure 3a), whereas a platelet-to-lymphocyte ratio (PLR ≥ 150) was not significantly associated (*p* = 0.129; Figure 3b). Neither baseline hemoglobin concentration nor serum squamous-cell carcinoma antigen (SCCA) showed prognostic relevance.

**Figure 4:**
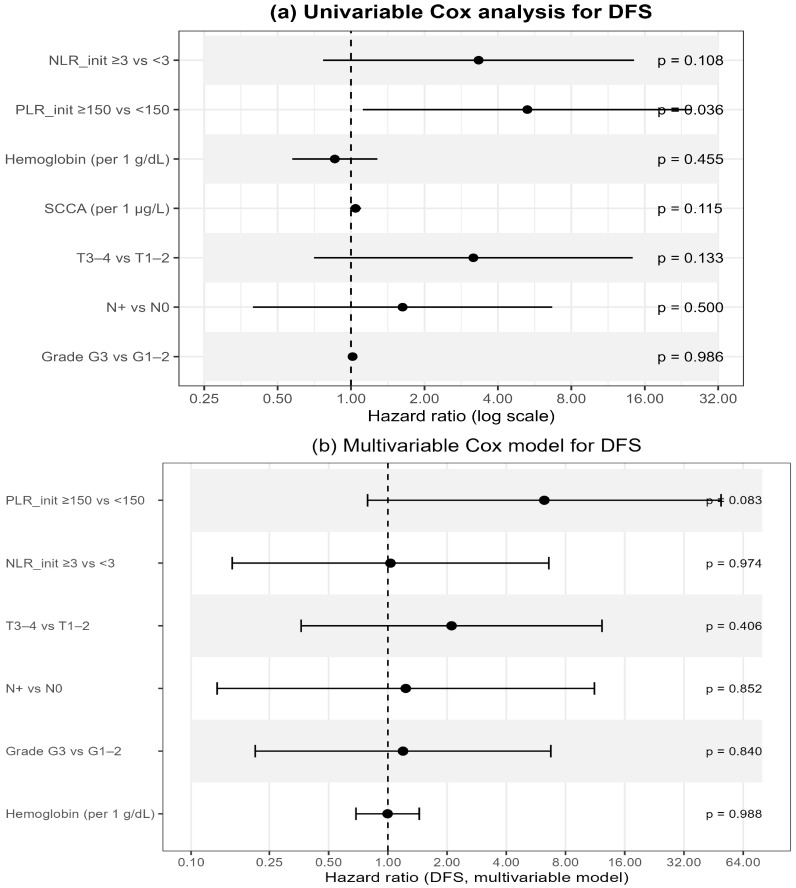
Multivariable Cox analysis for disease-free survival (DFS). (**a**) Univariable Cox analysis including baseline NLR, PLR, hemoglobin, SCCA, T-stage, N-stage, and tumor grade. (**b**) Multivariable Cox model integrating PLR, NLR, hemoglobin, T-stage, N-stage, and tumor grade. Points represent hazard ratios (HR) with 95% confidence intervals; the vertical dashed line marks HR = 1. *p*-values correspond to Wald tests. The number of DFS events, AIC, and concordance index (C-index) for each model are reported below the panels.

In univariable Cox regression (Table 7; Figure 4a), baseline PLR ≥ 150 was significantly associated with worse DFS (HR 5.28, 95% CI 1.12–24.97; *p* = 0.036), whereas NLR ≥ 3 showed only a borderline effect (HR 3.33, 95% CI 0.77–14.47; *p* = 0.108). Other hematologic or clinical variables—including hemoglobin, SCCA, T- and N-stage, and tumor grade—were not significant.

In the multivariable Cox model integrating hematologic and clinical variables (Table 7; Figure 4b), none of the factors retained independent significance. PLR ≥ 150 demonstrated a borderline association with poorer DFS (HR 6.24, 95% CI 0.79–49.38; *p* = 0.083), while all remaining variables showed non-significant effects. These findings suggest a potential—though exploratory—prognostic role of baseline systemic inflammatory markers, particularly PLR, as accessible indicators of tumor aggressiveness and host response.

Exploratory ROC-based analyses of alternative thresholds are summarized in Supplementary Table S1, where PLR ≥ 150 showed AUC 0.708 and a significant log-rank signal in screening, although it did not retain significance in the final Kaplan–Meier model (*p* = 0.129). Only baseline platelet counts above the cohort median (268 × 10^9^/L) remained significantly associated with DFS.

Because of the limited number of overall survival (OS) events (n = 4), OS analyses remain exploratory.

### 3.7. Exploratory Multivariable Models and Combined Risk Stratification

To further assess the prognostic value of hematologic indices, we developed exploratory models that combine nodal status with baseline NLR and PLR. In a multivariable Cox model including T category, nodal status, NLR, and PLR, only nodal positivity remained independently associated with disease-free survival (DFS), whereas hematologic parameters did not reach independent significance.

**Figure 5:**
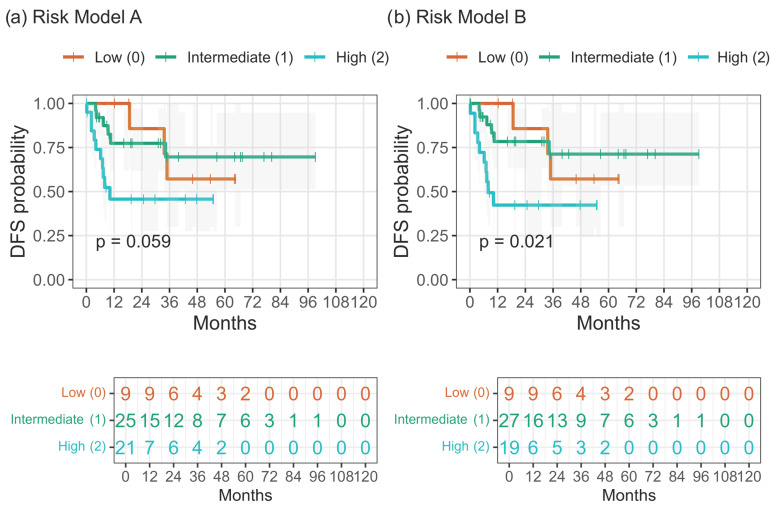
Disease-free survival (DFS) according to integrated risk models. (**a**) Model combining N status, PLR ≥ 150 and NLR ≥ 3 (score 0–3 grouped as Low = 0, Intermediate = 1, High = 2–3; *p* = 0.059). (**b**) Model combining N status and PLR ≥ 150 (score 0–2 grouped as Low = 0, Intermediate = 1, High = 2; *p* = 0.021). Kaplan–Meier curves show DFS probability with censored events (ticks). Shaded areas represent 95% confidence intervals; numbers at risk are shown below each panel.

**Figure 6:**
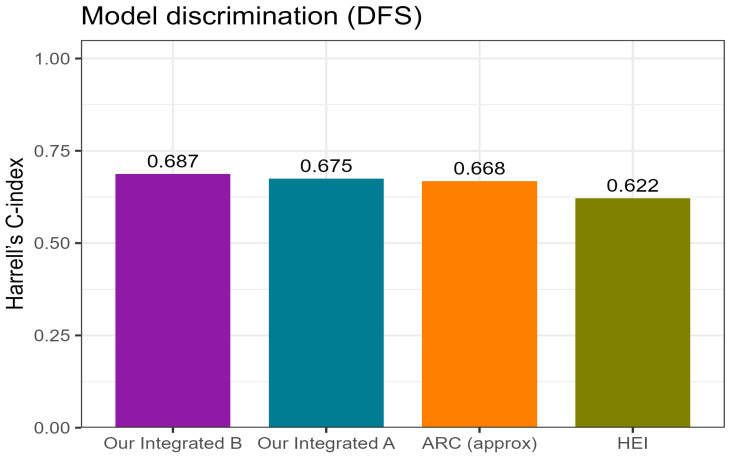
Comparison of model discrimination for disease-free survival (DFS). Harrell’s C-index values are shown for the proposed integrated models A and B, and for the ARC and HEI models. Higher C-index values indicate better discrimination.

When composite three-tier risk models were constructed, integration of nodal status with hematologic indices enhanced prognostic separation. Model A (N + PLR ≥ 150 ± NLR ≥ 3) showed a trend toward inferior DFS with increasing risk (*p* = 0.059), while the simplified Model B (N + PLR ≥ 150) achieved significant stratification (*p* = 0.021; Figure 5a,b; Table 8). Apparent discrimination by Harrell’s C-index was approximately 0.68–0.69 for the integrated models, with Model B slightly outperforming Model A and both exceeding the HEI and ARC classifiers (Figure 6). Internal resampling confirmed the same ranking of models (Figure S3).

Overall, these findings confirm that nodal status remains the dominant clinical determinant of prognosis, whereas incorporating baseline hematologic indices—particularly PLR—provides incremental prognostic value for DFS. Because of the very limited number of deaths (n = 4), OS analyses were regarded as exploratory and were not included in Table 8.

For clinical interpretation, the four evaluated indices represent related but conceptually distinct approaches. The HEI index reflects a composite baseline inflammatory profile driven by anemia, systemic immune–inflammation (SII), and eosinophilia. The ARC classifier integrates nodal status, hemoglobin, and SII, and was originally developed as a response-prediction tool for chemoradiotherapy. In contrast, our Integrated Models A and B anchor the inflammatory signal directly to nodal status and use only baseline PLR (±NLR) to create a simple, pragmatic three-tier score. Model A provides a more granular assessment by including both PLR and NLR, whereas Model B offers a streamlined two-parameter framework well suited for routine clinical use.

### 3.8. Comparative Performance and Model Utility

To benchmark the proposed integrated models against external hematology-based classifiers, their discrimination for disease-free survival (DFS) was compared with the Anal Cancer Response Classifier (ARC, approximation) and the Hemo-Eosinophil Inflammation (HEI) index (Figure 6; Table 9).

Both integrated models outperformed the external indices, achieving higher concordance (Harrell’s C-index ≈ 0.68–0.69 vs. 0.62–0.67 for HEI and ARC; Figure 6) and better overall model fit than N-only references (ΔAIC in favor of the integrated models; Supplementary Table S2). Among them, Model B (N + PLR ≥ 150) provided the most favorable balance between simplicity and predictive accuracy, whereas Model A (N + PLR ≥ 150 ± NLR ≥ 3) demonstrated comparable but slightly less consistent results.

Internal validation confirmed the stability and relative ranking of the models, with time-dependent AUC values ranging approximately from 0.60 to 0.78 across 12–60 months and IBS values around 0.18–0.22 up to 60 months (Figure S3; Table S2).

The correlation heatmap (Figure S4) showed only weak-to-moderate associations between hematologic indices and clinical variables, supporting their incremental—rather than redundant—prognostic value.

Taken together, integrating clinical nodal status with a simple baseline hematologic marker (PLR) enhances prognostic accuracy compared with previously published hematology-based indices, while maintaining interpretability and feasibility for routine clinical application.

## 4. Discussion

### 4.1. Overview and Key Findings

In our single-center cohort of 55 patients with anal squamous cell carcinoma (ASCC) treated with definitive chemoradiotherapy (CRT), overall survival (OS) remained excellent, showing a durable plateau beyond three years, whereas disease-free survival (DFS) declined more steeply to about 50% at five years. Most recurrences clustered within 24–36 months after treatment, reflecting patterns reported in population-based and IMRT-era cohorts [9,10].

Neither T nor N stage significantly influenced OS (Figures S1 and S2), though nodal positivity tended toward inferior DFS (Figure 2). These results align with prior evidence that local control and salvage therapy maintain OS even in node-positive disease [7,8,23]. Importantly, the limited prognostic separation achieved by conventional T and N stage contrasts with the substantially better discrimination observed for inflammation-based indices in our DFS analyses, underscoring the added value of these markers beyond anatomical staging.

### 4.2. Prognostic Value of Hematologic Indices

Systemic inflammatory markers are increasingly recognized as surrogates of host–tumor interactions in ASCC. In our analysis, baseline PLR ≥ 150 was associated with worse DFS in univariable Cox regression (HR 5.28, 95% CI 1.12–24.97; *p* = 0.036), but showed only a borderline effect in the multivariable model (*p* = 0.083) and non-significant separation in Kaplan–Meier curves (log-rank *p* = 0.129), whereas NLR ≥ 3 showed a weaker but directionally similar trend (log-rank *p* = 0.055; Cox *p* = 0.108; Figure 3 and Figure 4).

These findings are consistent with De et al. [15], who reported that high baseline NLR independently correlated with inferior OS and locoregional control, while treatment-related lymphopenia was not prognostic. Our observations suggest that the baseline inflammatory state, rather than transient cytopenia, better reflects long-term immune balance and tumor aggressiveness. Recent evidence further reinforces this concept. In a large multicentre analysis with accompanying meta-analysis, Wind et al. [25] demonstrated that several pre-treatment inflammatory markers, including NLR and PLR, were associated with survival in anal cancer, although effect sizes and optimal cut-offs varied across cohorts. Compared with that study, our work integrates PLR and NLR with nodal status into simple composite models and benchmarks them against established classifiers (HEI and ARC). Despite the smaller sample size, our results align with the broader literature and support the role of systemic inflammatory markers as an additional prognostic dimension that complements anatomical staging.

### 4.3. Integrated Clinical–Hematologic Models

Given the limited discrimination of single indices, we developed integrated models combining nodal status with baseline hematologic parameters. Importantly, the very limited separation observed for T and N stages in overall survival analyses is consistent with the low number of OS events in contemporary CRT-treated cohorts. By contrast, inflammation-based indices showed markedly better discrimination for disease-free survival, highlighting their potential value for prognostication in endpoints where event numbers allow meaningful separation.

Both the composite Model A (N + PLR ≥ 150 ± NLR ≥ 3) and the simplified Model B (N + PLR ≥ 150) stratified DFS into three risk tiers, with Model B offering the best balance of simplicity and predictive accuracy (*p* = 0.021; C-index ≈ 0.69) (Figure 5 and Figure 6).

When compared with external classifiers—the Anal Cancer Response Classifier (ARC) [16] and the Hemo-Eosinophil Inflammation (HEI) index [17,18]—both integrated models demonstrated higher discrimination and better model fit (Table S2).

Together, these results indicate that combining a single, robust inflammatory marker with nodal status provides pragmatic and clinically intuitive prognostic value for everyday practice.

### 4.4. Biological and Clinical Implications

The correlation heatmap (Supplementary Figure S4) confirmed strong interdependence among hematologic indices but only weak associations with clinical stage or radiotherapy (RT) parameters, supporting the notion that systemic inflammation represents a distinct biological axis. Elevated platelet and neutrophil–lymphocyte ratios may promote tumor progression through cytokine-driven angiogenesis, immune suppression, and microenvironmental remodeling—mechanisms increasingly implicated in ASCC biology [15,16,17,18].

Clinically, our results reaffirm nodal status as the principal prognostic determinant while showing that PLR adds incremental value for individualized risk assessment. Taken together with the weak OS stratification by T and N stage (Supplementary Figures S1 and S2), these findings highlight the complementary role of inflammation-based indices in refining prognostication where anatomical staging alone is insufficient. Such markers are low-cost, reproducible, and widely accessible, making them particularly suitable for real-world implementation or trial stratification.

Our toxicity profile mirrored large IMRT-based CRT series—frequent but manageable acute dermatitis, low rates of grade 3–4 gastrointestinal or genitourinary toxicity, and expected hematologic suppression, mainly lymphopenia—consistent with RTOG 0529 [22], Bazan et al. [23], Jethwa et al. [24], and Call et al. [19].

These results also parallel our group’s previous findings in hereditary breast cancer, where radiation-induced lymphopenia correlated with outcome, underscoring the link between immune function and treatment efficacy [26].

### 4.5. Strengths and Limitations

Strengths of this study include homogeneous treatment using modern IMRT-based CRT, standardized data collection, and comprehensive time-to-event analyses with transparent reporting of risk sets, global *p*-values, and concordance indices.

However, its retrospective single-center design introduces inherent bias, and the modest number of DFS events limits the precision of multivariable estimates. HPV status was unavailable for some patients, and ARC scoring required approximation. Baseline hematologic indices were derived from routine blood samples obtained at staging, prior to the start of chemoradiotherapy. Transient factors that may affect inflammatory markers (e.g., acute infections, recent systemic corticosteroid therapy, or underlying haematologic disorders) were not systematically recorded and could not be formally accounted for, which represents a limitation of this retrospective analysis.

Consequently, confidence intervals are wide, and the findings should be interpreted as hypothesis-generating. Nonetheless, their consistency with external cohorts and biological plausibility supports both validity and clinical relevance.

### 4.6. Future Perspectives

Our data propose a feasible, integrated prognostic framework combining N status and baseline PLR as a surrogate of systemic inflammation. These directions are consistent with the recent multicentre analysis by Wind et al. [25], which highlighted both the prognostic relevance and the methodological limitations of single inflammatory markers and emphasized the need for simple, clinically applicable composite models accompanied by prospective validation in standardized settings. Because conventional staging alone offers limited prognostic resolution—particularly for OS in early-stage disease—the integration of systemic inflammatory markers may help identify biologically higher-risk patients within the same anatomical stage. Future studies should pursue prospective, multicentre validation with standardized hematologic sampling, HPV stratification, and marrow-sparing RT techniques [19,20,21,22]. Incorporating immune and molecular biomarkers could further refine risk stratification and identify candidates for treatment de-escalation or immunotherapy integration.

In routine practice, inflammation-based indices may help identify patients who could benefit from closer post-treatment surveillance or early supportive interventions, particularly within the first 24–36 months when most recurrences occur. Although these markers do not currently guide therapeutic escalation, they may complement anatomical staging when deciding follow-up intensity or allocating patients to risk-adapted clinical studies.

From a translational standpoint, inflammation-based indices may also help to operationalize emerging biologically oriented treatment strategies. Elevated baseline NLR and PLR reflect a systemic pro-inflammatory and relatively lymphodepleted state that has been associated with poorer outcomes after CRT and could therefore identify patients at higher biological risk. Ongoing CRT–immunotherapy trials, such as RADIANCE (CRT ± durvalumab) [27] and EA2165 (CRT ± nivolumab) [28], aim to enhance locoregional and systemic control in such higher-risk groups. In parallel, the UK PLATO–ACT4 trial evaluating reduced-dose chemoradiotherapy for favourable-risk disease explores RT de-escalation where appropriate [29]. Once validated prospectively, simple composite indices such as our integrated models could support stratification of patients towards treatment intensification versus de-escalation, or enrolment into biologically stratified clinical studies, rather than serving as stand-alone determinants of therapy.

Overall, this work underscores the clinical relevance of inflammatory markers in ASCC and provides an accessible path toward personalized, risk-adapted patient management.

## 5. Conclusions

Definitive chemoradiotherapy (CRT) delivered using modern IMRT techniques achieved excellent overall survival in this real-world cohort of anal squamous cell carcinoma (ASCC), while disease-free survival was limited mainly by early relapses within the first three years after treatment.

Nodal positivity remained the dominant prognostic factor, whereas baseline hematologic indices—particularly an elevated platelet-to-lymphocyte ratio (PLR ≥ 150)—provided complementary insights into systemic inflammation and recurrence risk.

When integrated with nodal status, these simple composite clinical–hematologic models yielded clear three-tier separation of DFS outcomes and outperformed previously published inflammation-based indices in discriminatory accuracy. Such models are cost-free, reproducible, and readily implementable, relying solely on routinely collected clinical and laboratory data.

These findings underscore the prognostic relevance of systemic inflammation in ASCC and support the use of pragmatic, bedside-applicable composite models to refine risk assessment beyond anatomic staging. In clinical practice, these models may guide risk-adapted post-CRT surveillance—enabling intensified follow-up for high-risk patients during the first 24–36 months—and inform stratification strategies in future clinical trials.Prospective, multi-institutional validation with standardized hematologic sampling and HPV testing is warranted before these models can be incorporated into therapeutic decision-making, particularly for treatment escalation or de-escalation strategies.

## Figures and Tables

**Table 1:** Patient and treatment characteristics.

Characteristic	n (%)
Total patients	55 (100)
Age, years (median, range)	63 (37–78)
Sex	
Female	43 (78)
Male	12 (22)
AJCC T stage (8th ed.)	
T1	5 (9)
–T2	21 (38)
T3	19 (35)
–T4	10 (18)
AJCC N stage (8th ed.)	
N0	13 (24)
–N1a	9 (16)
N1b	7 (13)
N1c	10 (18)
–N1	15 (27)
–NX	1 (2)
Radiotherapy schema	
–SIB	24 (44)
RTOG	19 (35)
Sequential	12 (22)
RT duration, days (median, range)	42 (35–74)

**Table 2:** Survival outcomes.

Outcome	1-Year % (95% CI)	3-Year % (95% CI)	5-Year % (95% CI)	Median (Months, 95% CI)
**Overall survival (OS)**	96.2 (86.3–99.0)	90.0 (76.8–95.9)	90.0 (76.8–95.9)	NR
**Disease-free survival (DFS)**	68.0 (53.1–79.1)	55.6 (39.0–69.4)	51.0 (35.8–66.1)	84.0 (95% CI, not estimable)

Reverse Kaplan–Meier median post-RT follow-up: 53.1 months (95% CI 34.7–58.0).

**Table 3:** Patterns of failure among relapsed patients.

Relapse Type	N	%
Local recurrence (LR)	9	47.4
Distant—Pulmonary	6	31.6
Distant—Hepatic	3	15.8
Distant—Other	5	26.3
Combined local and distant	5	26.3
Summary of time-to-relapse (TTR) in months		
Metrics	Value	
Relapsers (N)	19 (34.5%)	
Median TTR (months)	7.3	
Range (months)	2.3–35	
IQR (months)	4.0–10.0	

**Table 4:** Acute and late toxicities (CTCAE v5.0).

Toxicity Type	Any Grade n (%)	Grade 3–4 n (%)
Acute		
Skin	53 (96.3%)	20 (36.4%)
Gastrointestinal	43 (78.2%)	0
Genitourinary	31 (56.4%)	0
Hematologic	48 (87.3%)	26 (47.3%)
Late (≥3 months)		
Skin	23 (43.4%)	0
Gastrointestinal	17 (31%)	1 (2%)
Genitourinary	14 (26%)	1 (2%)
Other	18 (34%)	3 (6%)

Note: Documentation of late non-hematologic toxicities was less consistent in early records; results should therefore be interpreted cautiously. Rates of acute skin toxicity reflect improved prophylaxis practices introduced in recent years, resulting in later onset, shorter duration, and good recovery.

**Table 5:** Univariable and multivariable Cox regression analyses for DFS.

Covariate	Univariable HR (95% CI)	*p*-Value	Multivariable HR (95% CI)	*p*-Value
T stage (T3–4 vs. T1–2)	1.42 (0.57–3.54)	0.455	1.05 (0.40–2.74)	0.925
Nodal status (N+ vs. N0)	2.79 (0.81–9.66)	0.105	2.74 (0.74–10.10)	0.130
HPV (positive vs. other)	0.83 (0.33–2.12)	0.697	–	–
Grade (G3 vs. G1–2)	1.12 (0.38–3.37)	0.835	–	–
Age per year increase	0.99 (0.95–1.03)	0.510	–	–

Note: Cox regression models for OS were not reliable due to the low number of events (n = 4) and are therefore not presented.

**Table 6:** Subgroup analyses of DFS (Kaplan–Meier estimates).

Grouping	Level	n	Events	12 m DFS %	36 m DFS %	60 m DFS %	*p* (Log-Rank)
T stage	≤T2	26	8	78.1%	59.9%	59.9%	0.401
	≥T3	29	11	62.5%	54.7%	54.7%	
Nodal	N0	13	3	100%	70.7%	70.7%	0.102
	N+	42	16	59.0%	53.6%	53.6%	
Age	≤70	37	12	73.0%	58.2%	58.2%	0.89
	>70	18	7	63.3%	54.3%	54.3%	
OTT (days)	≤49	47	16	72.3%	56.1%	56.1%	0.84
	>49	8	3	75.0%	68.8%	68.8%	
HPV	HPV+	37	12	69.7%	59.1%	59.1%	0.71
	Other/unk	18	7	76.2%	52.3%	52.3%	

Note: No subgroup reached statistical significance. Nodal positivity and higher T stage were associated with numerically inferior DFS, while age, overall treatment time, and HPV status showed no measurable impact.

**Table 7:** Cox regression analysis for disease-free survival (DFS).

Variable	HR (95% CI)	*p*-Value
Univariate analysis		
PLR_init ≥ 150 vs. <150	5.28 (1.12–24.97)	0.036
NLR_init ≥ 3 vs. <3	3.33 (0.77–14.47)	0.108
Hemoglobin (per 1 g/dL)	0.86 (0.57–1.28)	0.455
SCCA (per 1 µg/L)	1.04 (0.99–1.10)	0.115
T3–4 vs. T1–2	3.17 (0.70–14.28)	0.133
N+ vs. N0	1.63 (0.40–6.68)	0.500
Grade G3 vs. G1–2	1.01 (0.20–5.07)	0.986
Multivariable analysis		
PLR_init ≥ 150 vs. <150	6.24 (0.79–49.38)	0.083
NLR_init ≥ 3 vs. <3	1.03 (0.16–6.58)	0.974
T3–4 vs. T1–2	2.11 (0.36–12.26)	0.406
N+ vs. N0	1.23 (0.14–11.22)	0.852
Grade G3 vs. G1–2	1.19 (0.21–6.74)	0.840
Hemoglobin (per 1 g/dL)	1.00 (0.69–1.44)	0.988

**Table 8:** Integrated prognostic models combining nodal status and hematological indices (disease-free survival).

Model	Groups	Global Log-Rank *p*	C-Index (Cox)	HR (95% CI)	Comment
Integrated A: N + PLR ≥ 150 ± NLR ≥ 3	Low = 9 Intermediate = 25 High = 21	0.059	0.675	2.63 (0.97–7.16)	Trend toward significant stratification
Integrated B: N + PLR ≥ 150	Low = 9 Intermediate = 27 High = 19	0.021	0.687	2.88 (1.10–7.53)	Significant prognostic separation

Notes: HRs are interpreted per one-step increase in the integrated risk score (Low → Intermediate → High). Apparent C-indices correspond to Figure 6; bootstrap—and IPCW-validated metrics, as well as time-dependent AUC and IBS values, are presented in Figure S3.

**Table 9:** Comparative performance of integrated and external hematology-based models for disease-free survival (DFS).

Model	Risk Group Definition	Groups (n)	Global Log-Rank *p*	C-Index (Cox)	HR (High vs. Low) (95% CI)	Comment
Integrated A (N + PLR ≥ 150 ± NLR ≥ 3)	Three-tier (Low = 0; Intermediate = 1; High = ≥ 2)	Low = 9; Int = 25; High = 21	0.059	0.675	2.63 (0.97–7.16)	Trend toward stratification
Integrated B (N + PLR ≥ 150)	Three-tier (Low = 0; Intermediate = 1; High = 2)	Low = 9; Int = 27; High = 19	0.021	0.687	2.88 (1.10–7.53)	Significant separation
ARC (approx)	N+, Hb < 12 g/dL, SII > 560 (0–3 points)	Low = 14; Int = 25; High = 16	0.067	0.668	2.19 (0.81–5.90)	Borderline
HEI [17]	Hb < 12 g/dL + SII > 560 + Eos ≥ 100/µL (0–3 points)	Low = 31; High = 24	0.081	0.622	1.94 (0.73–5.16)	Borderline

Notes: Higher C-index and lower AIC indicate superior performance. Apparent C-indices correspond to Figure 6 and Table 9; optimism-corrected C-indices, together with time-dependent AUC and integrated Brier score (IBS ≤ 60 months), are provided in Figure S3 and Table S2. OS benchmarking was not performed due to the very limited number of deaths (n = 4).

## Data Availability

The data presented in this study are not publicly available due to patient privacy and institutional ethical restrictions. De-identified data may be made available upon reasonable request to the corresponding author, subject to institutional approval.

## Supplementary Materials

The following supporting information can be downloaded at: https://www.mdpi.com/article/10.3390/cancers17233838/s1. Figure S1. Overall survival according to T stage. Kaplan–Meier curves for overall survival (OS) stratified by T stage (T1–2 vs. T3–4). Shaded areas represent 95% confidence intervals. The table below shows the number of patients at risk at 12-month intervals. No significant difference in OS was observed (log-rank *p* = 0.484). Figure S2. Overall survival according to N stage. Kaplan–Meier curves for OS stratified by nodal status (N0 vs. N+). Shaded areas represent 95% confidence intervals. The number of patients at risk is shown at 12-month intervals. No significant difference was observed (log-rank *p* = 0.275). Figure S3. Model validation and performance comparison for disease-free survival (DFS). (a) Bootstrapped discrimination (Harrell’s C-index, median ± 95% CI); (b) Time-dependent AUC at 12, 36 and 60 months; (c) Prediction-error curves with integrated Brier score (IBS ≤ 60 months). Integrated models (A and B) demonstrated superior discrimination and calibration compared with external classifiers (ARC and HEI). Figure S4. Spearman correlation heatmap of hematologic and clinical parameters. The matrix shows pairwise Spearman correlation coefficients (ρ) among baseline NLR, PLR, platelet count, and clinical variables (T stage, N status, tumor grade, and radiotherapy length). Only statistically significant correlations (*p* < 0.05) are labelled. Table S1. Prognostic performance of hematologic indices for disease-free survival (DFS). Univariable log-rank and ROC-based analyses of inflammatory markers, including NLR, PLR, platelet count, hemoglobin, and leukocyte subsets. Literature and Youden-derived cut-offs, classification metrics, and associations with DFS are presented. Table S2. Validation metrics for integrated prognostic models compared with external classifiers (HEI and ARC). Metrics include global log-rank *p*-values, optimism-corrected Harrell’s C-indices (95% CI), ΔAIC relative to the N-only reference model, time-dependent AUC at 36 months, and integrated Brier score (IBS ≤ 60 months). Values correspond to Supplementary Figure S3.

## Abbreviations

**5-FU**	5-Fluorouracil
**AIC**	Akaike’s Information Criterion
**AJCC**	American Joint Committee on Cancer
**ARC**	Anal Cancer Response Classifier
**ASCC**	Anal squamous cell carcinoma
**CI**	Confidence interval
**CRT**	Chemoradiotherapy
**CTCAE**	Common Terminology Criteria for Adverse Events
**DFS**	Disease-free survival
**EORTC 22861**	European Organisation for Research and Treatment of Cancer trial 22861
**ESTRO ACROP**	European Society for Radiotherapy and Oncology—Advisory Committee on Radiation Oncology Practice
**HEI**	Hemo-Eosinophil Inflammation index
**HPV**	Human papillomavirus
**HR**	Hazard ratio
**IARC**	International Agency for Research on Cancer
**IGRT**	Image-guided radiotherapy
**IMRT**	Intensity-modulated radiotherapy
**MMC**	Mitomycin C
**MVCT**	Megavoltage computed tomography
**NLR**	Neutrophil-to-lymphocyte ratio
**OS**	Overall survival
**OTT**	Overall treatment time
**PLR**	Platelet-to-lymphocyte ratio
**RTOG 98-11**	Radiation Therapy Oncology Group trial 98-11
**RTOG**	Radiation Therapy Oncology Group
**SII**	Systemic immune-inflammation index
**UKCCCR ACT I**	United Kingdom Coordinating Committee on Cancer Research Anal Cancer Trial I
**ACT II**	Anal Cancer Trial II
