# Supplementary material for: Prognostic Value of Hematologic Indices and Composite Models in Anal Squamous Cell Carcinoma Treated with Image-Guided Chemoradiotherapy
Source: Cancers (Basel). 2025 Nov 29;17(23):3838. doi: 10.3390/cancers17233838 (PMC12691201; doi:10.3390/cancers17233838)
Supplement: Supplementary file 1 [file cancers-17-03838-s001.zip › cancers-3983781-supplementary.pdf]

Supplementary Figures and Tables

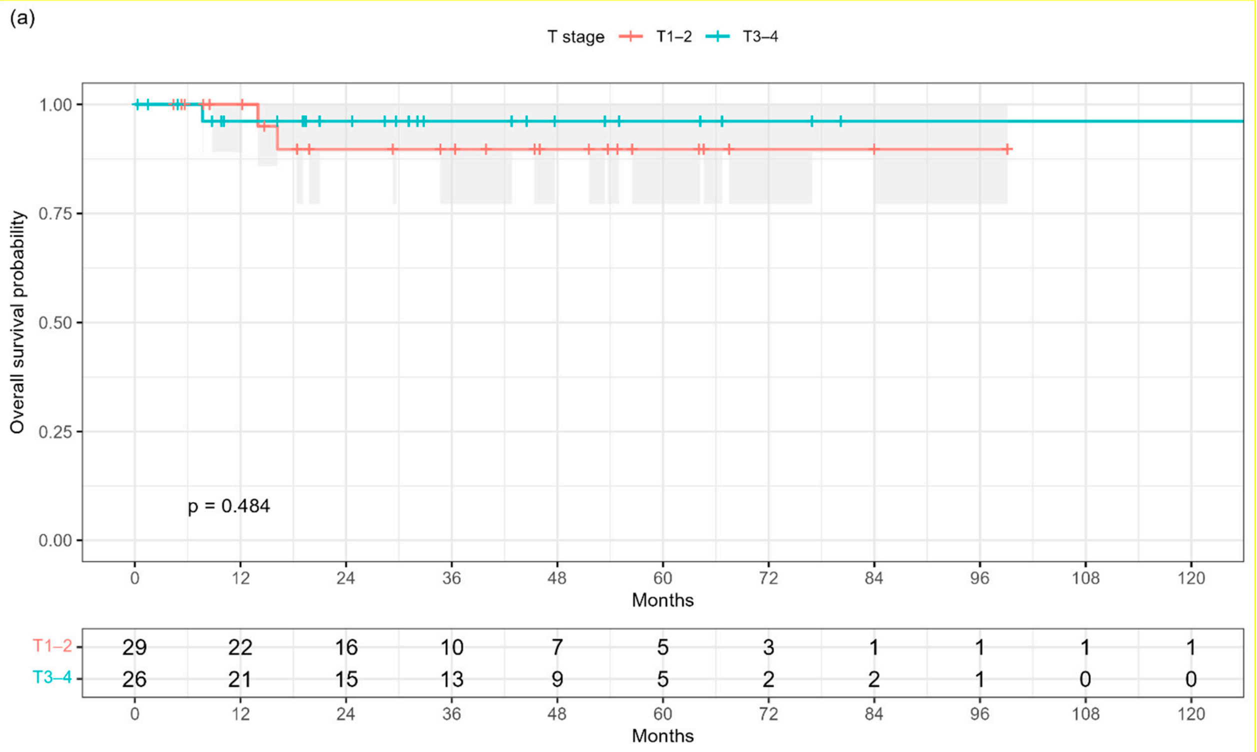

Figure S1. Overall survival according to T stage.

Kaplan–Meier curves for overall survival (OS) stratified by T stage (T1–2 vs. T3–4). Shaded areas represent 95 % confidence intervals. The table below shows the number of patients at risk at 12-month intervals. No significant difference in OS was observed between groups (log-rank p = 0.484).

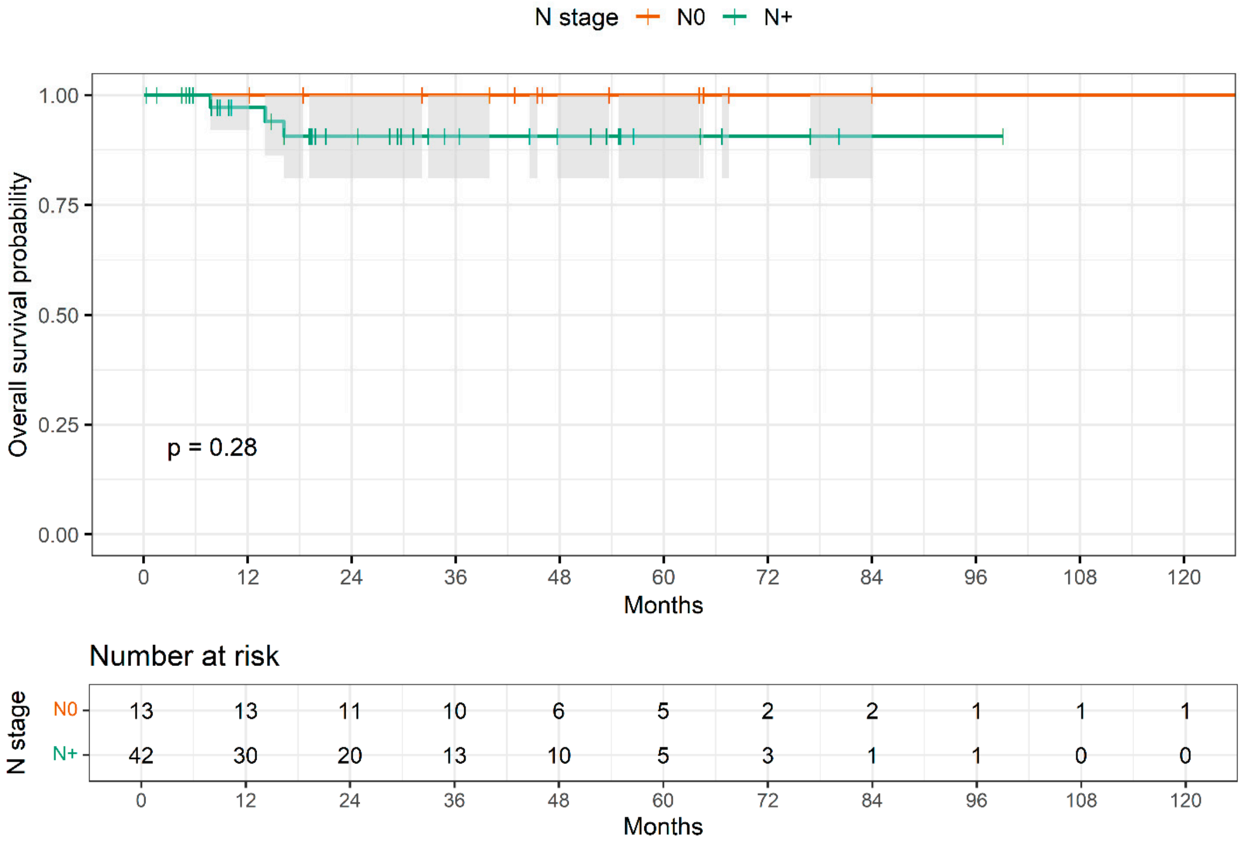

Figure S2. Overall survival according to N stage.

Kaplan–Meier curves for overall survival (OS) stratified by nodal status (N0 vs. N+). Shaded areas represent 95 % confidence intervals. The table below shows the number of patients at risk at 12-month intervals. No significant difference in OS was observed between groups (*log-rank p = 0.275*).

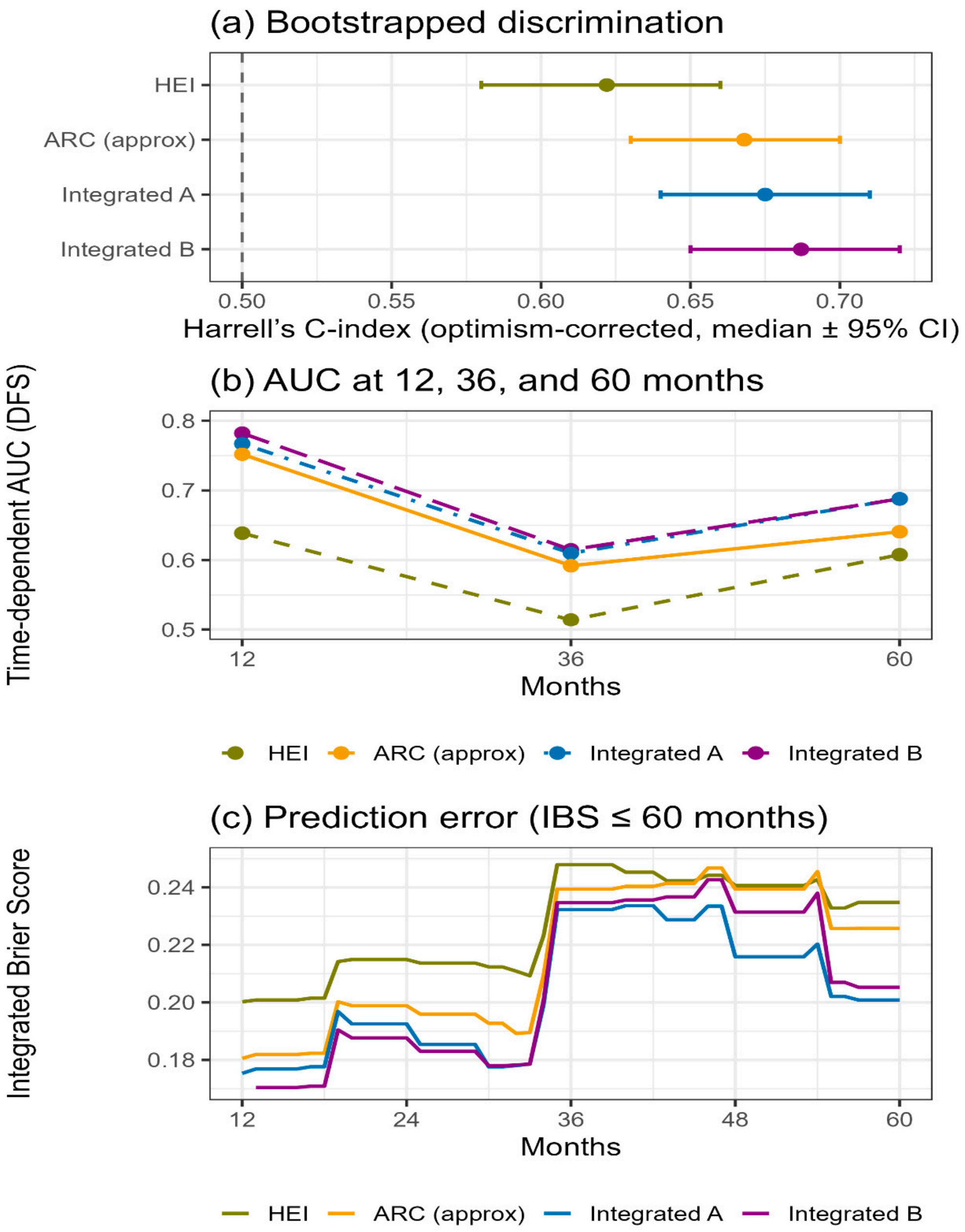

Figure S3. Model validation and performance comparison for disease-free survival (DFS).
(a) Bootstrapped discrimination (Harrell's C-index, median ± 95 % CI).
(b) Time-dependent AUC at 12, 36 and 60 months.
(c) Prediction-error curves with integrated Brier score (IBS ≤ 60 months).
The integrated risk models (A and B) outperformed external classifiers (ARC and HEI) in discrimination and calibration performance, confirming consistent internal validity.

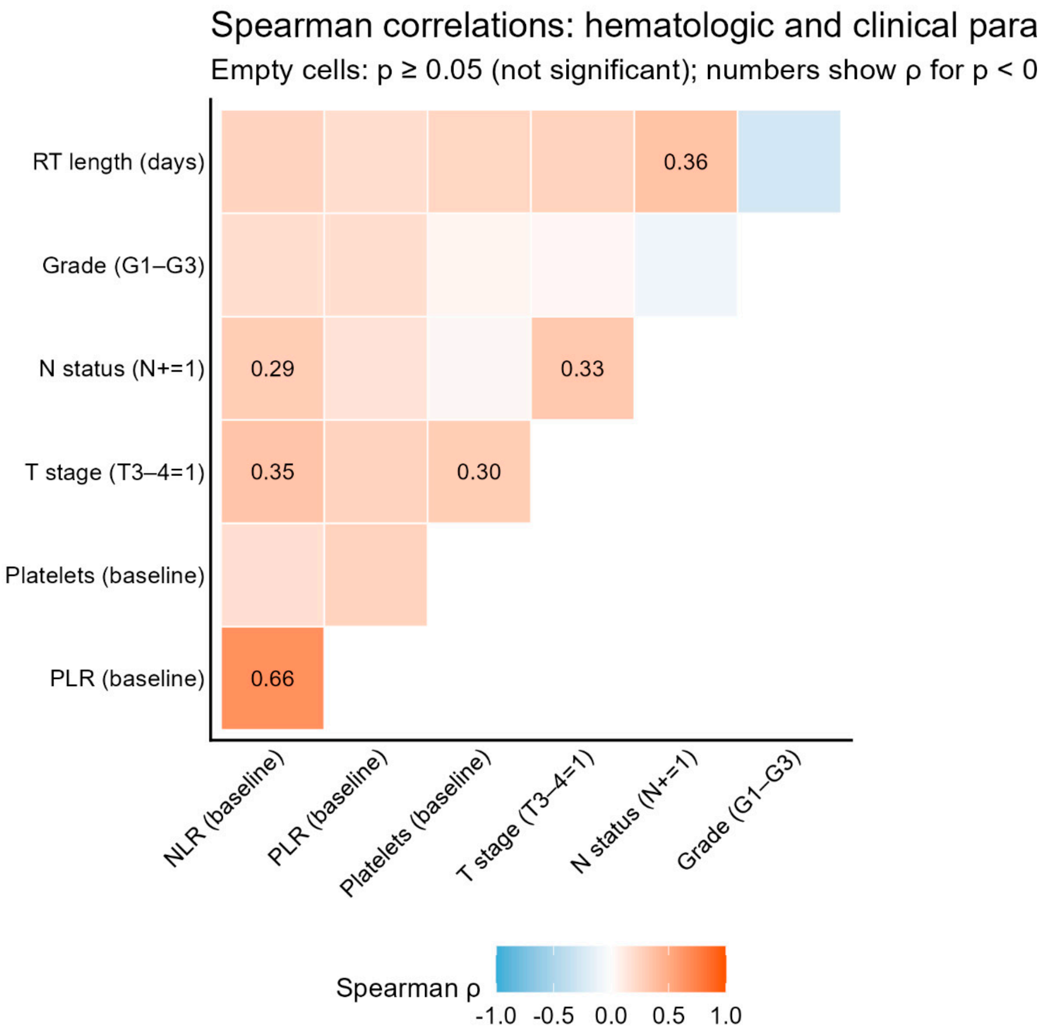

Figure S4. Spearman correlation heatmap of hematologic and clinical parameters.
The matrix shows pairwise Spearman's correlation coefficients (ϱ) among baseline NLR, PLR, platelet count, and clinical variables (T stage, N status, tumor grade, and radiotherapy length). Numbers are displayed only for statistically significant pairs ($p < 0.05$, two-sided); empty cells denote non-significant associations. Colors indicate the direction and magnitude of ϱ (blue = negative, orange = positive). *p*-values are unadjusted for multiple comparisons.

Table S1. Prognostic performance of hematologic indices for disease-free survival (DFS).

Univariable log-rank and ROC-based analyses of candidate hematologic indices were performed to identify potential prognostic cut-offs. Among the evaluated markers, baseline platelet-to-lymphocyte ratio (PLR) showed the highest discriminatory accuracy (AUC = 0.708, optimal Youden cut-off = 186.3, $p < 0.05$). However, when dichotomized at the literature threshold of 150, PLR did not retain statistical significance in the final Kaplan–Meier analysis ($p = 0.129$; Figure 3b). Similarly, a higher neutrophil-to-lymphocyte ratio (NLR ≥ 3) demonstrated only a borderline association with inferior DFS ($p = 0.055$; Figure 3a). Other parameters—including platelet count, hemoglobin concentration, and leukocyte subsets—showed no consistent prognostic signal, except for baseline platelet count above the cohort median (268 × 10^9/L), which remained significantly associated with shorter DFS. These findings suggest that while inflammatory ratios such as PLR and NLR capture systemic response patterns, their prognostic relevance in ASCC remains exploratory.

Marker	Phase	Decision Rule	Youden Cut-Off	Literature Cut-Off	Sensitivity	Specificity	Youden Index	AUC	Association with DFS
NLR (init)	init	x ≥ threshold	1.75	3	0.875	0.472	0.347	0.632	Trend ($p \approx 0.074$)

NLR (nadir)	nadir	x ≤ threshold	11.88	3	1.000	0.371	0.371	0.648	NS
NLR (post)	post	x ≤ threshold	11.88	3	1.000	0.257	0.257	0.498	NS
PLR (init)	init	x ≥ threshold	186.3	150	0.625	0.861	0.486	0.708	Significant (log-rank p = 0.0026)
Platelet count (baseline)	init	x ≥ cohort median	-	-	-	-	-	-	Significant (DFS)
PLR (nadir)	nadir	x ≤ threshold	406.3	150	0.500	0.735	0.235	0.568	NS
PLR (post)	post	x ≤ threshold	877.3	150	1.000	0.229	0.229	0.545	NS
SCCA (init)	init	x ≥ threshold	2.30	-	0.714	0.719	0.433	0.708	NS
Hemoglobin (init)	init	x ≤ threshold	126	-	0.625	0.806	0.431	0.635	NS

Note: While PLR ≥ 150 showed statistical significance in the initial ROC–Youden screening (p = 0.0026), it was not significant in the final Kaplan–Meier analysis (p = 0.129; Figure 3b).
The platelet cohort median at baseline was 268 × 10^9/L; the row "Platelet count (baseline)" applies the decision rule x ≥ median.
Youden-derived cut-offs and classification metrics were obtained from ROC analyses on DFS events. Literature cut-offs were included where established (e.g., NLR ≥ 3, PLR ≥ 150). ROC–Youden screening evaluates optimal cut-offs independently of literature thresholds; therefore, significance observed for PLR at the Youden-derived cut-off (186.3) does not imply significance for the predefined clinical cut-off of 150 used in the main Kaplan–Meier and Cox analyses.
NS = not significant; AUC = area under the ROC curve.

Table S2. Validation metrics for integrated prognostic models compared with external HEI and ARC classifiers (DFS).

Median optimism-corrected Harrell's C-indices with 95 % confidence intervals were obtained using 1 000-fold bootstrap resampling. Time-dependent AUC and integrated Brier score (IBS ≤ 60 months) were computed using inverse probability of censoring weighting (IPCW). ΔAIC values are reported relative to the nodal-status-only reference model.
Apparent (non-corrected) C-indices from Cox models are shown in Table 9 and Figure 6 for comparability with standard clinical reporting. The values reported in Table S2 therefore represent optimism-corrected internal validation metrics and may differ slightly—but consistently—from the apparent C-indices shown in Table 9.
Values correspond to those shown in Supplementary Figure S3.

**Model**	**Global Log-Rank *p***	**C-Index (95% CI)**	**ΔAIC vs N-Only**	**AUC @36m**	**IBS ≤60m**	**Comment**
Integrated B (N + PLR ≥ 150)	0.021	0.693 (0.576–0.787)	-3.2	0.615	0.202	Best overall discrimination
Integrated A (N + PLR ≥ 150 ± NLR ≥ 3)	0.059	0.695 (0.569–0.805)	-2.7	0.610	0.199	Stable performance
ARC (approximation)	0.067	0.659 (0.495–0.760)	-1.8	0.592	0.212	Moderate separation
HEI (Rimini et al., 2021)	0.081	0.596 (0.464–0.709)	0	0.514	0.222	Lowest discrimination

Note: Higher C-index and lower AIC indicate superior model performance. AUC = area under the curve; IBS = integrated Brier score; CI = confidence interval; ΔAIC = change in Akaike information criterion relative to the N-only reference model.
